# *Sogatella furcifera* Saliva Mucin-like Protein Is Required for Feeding and Induces Rice Defences

**DOI:** 10.3390/ijms23158239

**Published:** 2022-07-26

**Authors:** Yudi Liu, Jinyu Yi, Haokang Jia, Yutong Miao, Maolin Hou

**Affiliations:** State Key Laboratory for Biology of Plant Diseases and Insect Pests, Institute of Plant Protection, Chinese Academy of Agricultural Sciences, No. 2, West Yuan Ming Yuan Road, Beijing 100193, China; yjy518898@163.com (J.Y.); jiahaokang6217@163.com (H.J.); miaoyutong@bjfu.edu.cn (Y.M.)

**Keywords:** *Sogatella* *furcifera*, mucin-like protein, salivary proteins, plant-insect interaction, rice defense response

## Abstract

The white-backed planthopper (WBPH), *Sogatella furcifera*, is one of the most important piercing-sucking pests of rice (*Oryza sativa*) in Asia. Mucin-like salivary protein (SFMLP) is highly expressed in the salivary glands of WBPH, which plays an important role in WBPH feeding. In this study, WBPH injected with *dsSFMLP* had difficulty in sucking phloem sap from rice plants, which significantly reduced their food intake, weight, and survival. In contrast, the knockdown of the *SFMLP* gene had only a marginal effect on the survival of WBPH fed an artificial diet. Further studies showed that silencing *SFMLP* resulted in the short and single-branched salivary sheaths secretion and less formation of salivary flanges in rice. These data suggest that *SFMLP* is involved in the formation of the salivary sheath and is essential for feeding in WBPH. Overexpression of the *SFMLP* gene in rice plants promoted the feeding of WBPH, whereas silencing the gene in rice plants significantly decreased WBPH performance. Additionally, it was found that overexpression of *SFMLP* in rice plants elicited the signalling pathway of SA (salicylic acid) while suppressing JA (jasmonic acid); in contrast, silencing of the *SFMLP* gene in rice plants showed the opposite results. This study clarified the function of *SFMLP* in WBPH feeding as well as mediating rice defences.

## 1. Introduction

Plants and piercing-sucking pests have been engaged in a coevolutionary arms race in which herbivore saliva plays a significant role. Herbivores secrete salivary effectors into host plant cells to facilitate feeding, whereas plants recognize attackers and activate complex defence responses when attacked by herbivores [1,2].

Effectors produced by herbivores, which are proteins or other molecules, can affect host plant structures and functions [3]. Several effectors have been identified in *Nilaparvata lugens* (Stål), including *β*-1,3-glucanase [4], endo-*β*-1,4-glucanase [5], EF-hang calcium-binding protein [6], and mucin-like protein [7,8]. Activation of the effector aids *N. lugens* in overcoming the rice cell wall defence and facilitates continuous phloem feeding. Three effectors, i.e., vitellogenin [9], calcium-binding protein [10], and DNase II [11], reported in *Laodelphax striatellus* (Fallen), can improve its performance and attenuate rice defences when they are secreted into rice plants. In aphids, effectors have also been identified, such as CathB3, C002, Me10, Me23, Mp2, and Mp55, which aid in aphid survival and regulate host defence [12,13,14,15,16,17].

The defence responses of plants to phloem-sucking insect pests usually induce salicylic acid (SA) accumulation and suppression or only modest induction of jasmonic acid (JA) defences [18,19]. There are several functional genes that have been identified for the biosynthesis of phytohormones in inducing defence responses. For example, the *NPR1* (non-expressor of pathogenesis-related genes 1) and *ICS1* (isochorismate synthase 1) genes are reported to be SA synthesis-related genes, and *AOS2* (allene oxide synthase 2) and *LOX* (lipoxygenase) genes are involved in JA biosynthesis [11,20,21]. For instance, the gene *Bt56*, which is a whitefly salivary protein, promotes whitefly performance on tobacco and induces the SA signalling pathway in host plants [21]. The infestations of whiteflies and aphids on *Arabidopsis thaliana* reduce or only modestly induce JA defences but induce SA signalling pathway genes [22,23,24]. Activation of the SA pathway can improve the whitefly performance on tobacco and suppression of the JA signalling pathway [25]. The infestation of the white-backed planthopper (WBPH), i.e., *Sogatella furcifera,* increases SA-mediated rice plant defences, and the infection of the southern rice black-streaked dwarf virus in the WBPH also can upregulate SA expression levels to a lesser extent [20].

WBPH is a severe phloem-sucking rice pest in Asia, which can cause serious damage by feeding on rice sap and transmitting viruses. During feeding, WBPH stylets penetrate rice cell walls, and their salivary glands secrete both gelling and watery saliva into plant. The gelling saliva can quickly form a complete salivary sheath in the plant, which encases the full length of the stylet and provides mechanical stability and protection for WBPH feeding. In the transcriptome analysis of the WBPH salivary gland, the mucin-like protein gene (*SFMLP*) was found to be highly expressed in WBPH salivary glands. In insects, several salivary mucin-like proteins have been identified. A mucin-like protein in the *Anopheles gambiae* salivary glands helps it lubricate its mouthparts [26]. The salivary mucin-like protein of *N. lugens* plays an important role in the formation of its salivary sheath and feeding on host plants, and it is involved in plant–insect interactions [7,8]. However, the functions of SFMLP in WBPH are largely unknown.

Expression of double-stranded RNA (*dsRNA*), which is designed against important insect genes, in transgenic plants has provided protection against pests [27]. In recent years, expression of *d*s*RNA* of target genes in transgenic plants has been applied extensively against sap-sucking pests such as aphids [28,29,30], whiteflies [21,27], and *N. lugens* [31,32]. These results revealed that the salivary effectors played essential roles on the suppression of host plant immunity and promotion of insect performance. Therefore, these effectors would be the potential targets for phloem-sucking pest control by employing the transgenic plant-mediated RNAi technology.

Here, our results demonstrate that *SFMLP* is necessary for the formation of salivary sheath and feeding of WBPH on host rice plants. We report that overexpression of *SFMLP* in rice plants enhances WBPH performance and induces the SA signalling pathway, whereas silencing of *SFMLP* decreases WBPH performance and impairs the SA signalling pathway. All the results provide valuable aid for developing environmentally friendly, targeted control methods for WBPH.

## 2. Results

### 2.1. SFMLP Is Highly Expressed in WBPH Salivary Glands

The full-length cDNA of *SFMLP* contains a 2160 bp open-reading frame, which encodes 719 amino acid residues. SignalP- and TMHMM-predicted SFMLP constituted the signal peptide, with a likely cleavage site between residues 19 and 20. SFMLP has a high proportion of Ser (22.8%).

### 2.2. Bioassay of WBPH Injection with dsSFMLP

**Figure 1:**
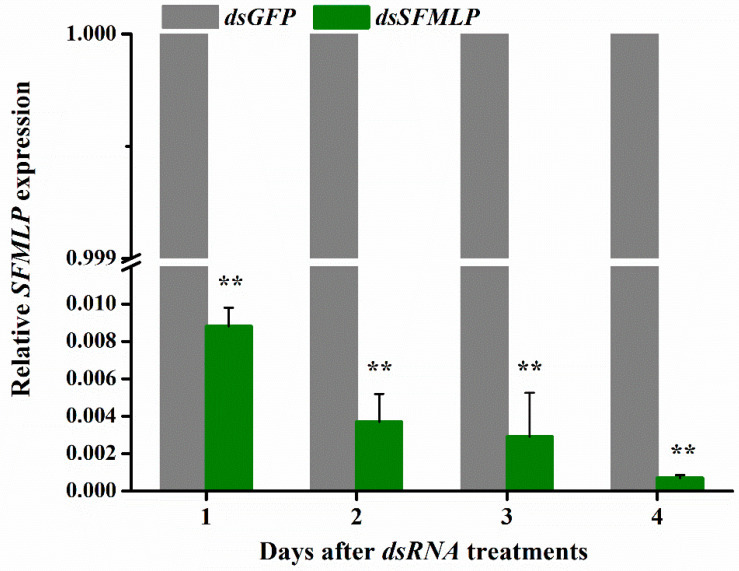
Relative expression level of *SFMLP* in WBPH after injection of *dsRNA.* ds*GFP* indicates WBPH injected with *GFP*-*dsRNA*; *dsSFMLP* indicates WBPH injected with *SFMLP*-*dsRNA*. The mRNA expression level in the *dsGFP* group is designated as 1.0. ** indicates statistically significant differences (*t-*test: *p* < 0.01). Bars, ±SEM.

Double-stranded RNA (*dsRNA*) of *SFMLP* was synthesized and injected into 5th instar WBPH nymphs to conduct RNA interference (RNAi) experimental treatment. The results revealed that *dsSFMLP* showed a very strong silence effect, and it decreased the *SFMLP* transcript levels significantly (>99%) from the 1st day after treatment compared with the levels of WBPH injection with *dsGFP* (Figure 1).

**Figure 2:**
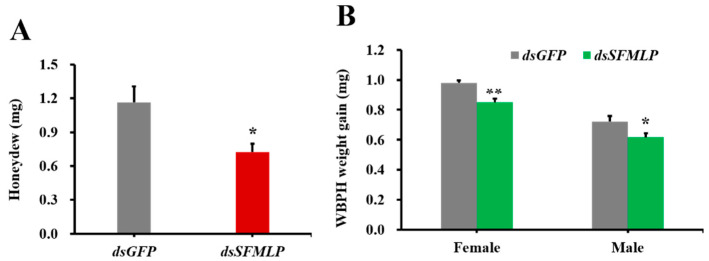
Effects of *SFMLP* silencing on WBPH performance. (**A**) The excreted honeydew of WBPH after injection of *dsRNA*; (**B**) the body weight gains of newly emerged WBPH females and males injected with *dsRNA*. *dsGFP* indicates WBPH injected with *GFP*-*dsRNA*; *dsSFMLP* indicates WBPH injected with *SFMLP*-*dsRNA*. * and ** indicate statistically significant differences (*t-*test: *p* < 0.05 and *p* < 0.01). Bars, ±SEM.

The amount of honeydew excreted by WBPH was significantly reduced after silencing of *SFMLP*, with levels only 62.2% of those with silenced *dsGFP* (Figure 2A). The body weights of WBPH females and males injected with *dsSFMLP* both showed significant differences from those injected with *dsGFP* (Figure 2B). The body weights of new emerging females and males with silenced *SFMLP* were 86.74% and 85.46% of those of females and males with silenced *GFP*, respectively (Figure 2B).

### 2.3. The Effects of SFMLP Knockdown on Feeding Behavior

**Figure 3:**
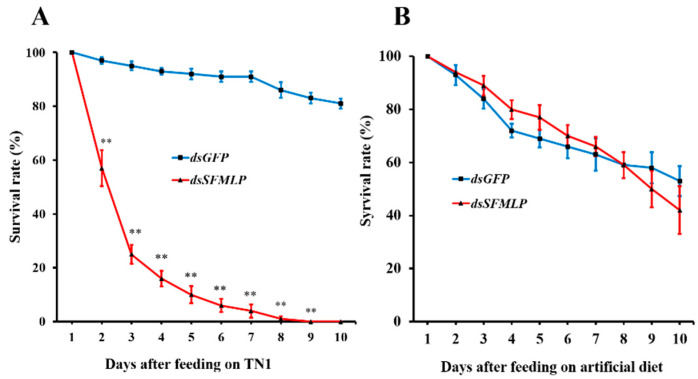
Effects of *SFMLP* knockdown on the survival rates of WBPH. ds*GFP* indicates WBPH injected with *GFP*-*dsRNA*; *dsSFMLP* indicates WBPH injected with *SFMLP*-*dsRNA*. (**A**) The survival rates of WBPH feeding on TN1; (**B**) the survival rates of WBPH feeding on artificial diet. “**” indicates statistically significant differences (*t* test: *p* < 0.01). Bars, ±SEM.

To investigate the role of *SFMLP* on feeding, we compared the survival rates within 1–10 days for WBPH developed from the 2nd–3rd instar nymphs between feeding on TN1 rice and the artificial diet in Parafilm sachets. The survival rates of WBPH nymphs with silenced *SFMLP* raised on TN1 rice decreased significantly on the 2nd day after injection, dropped to 20% on the 4th day after injection, and the nymphs died completely on the 9th day after injection compared with the *dsGFP* control group (Figure 3A). On the contrary, there was no significant difference in the survival rates within 1–10 days between WBPH developed from the 2nd–3rd instar nymphs with silenced *SFMLP* and the *dsGFP* control group raised on the artificial diet (Figure 3B). These results reveal that *SFMLP* is important to feeding.

**Figure 4:**
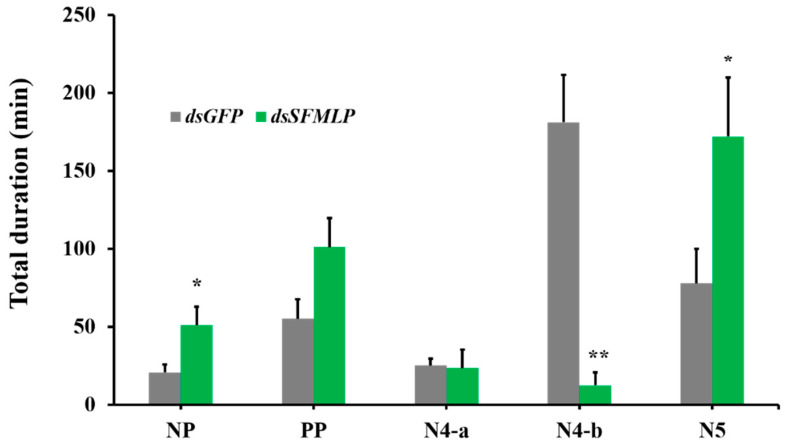
Effects of *SFMLP* knockdown on feeding behaviour of WBPH ds*GFP* indicates WBPH injected with *GFP*-*dsRNA*; *dsSFMLP* indicates WBPH injected with *SFMLP*-*dsRNA*. * and ** indicate statistically significant differences (*t-*test: *p* < 0.05 and *p* < 0.01). Bars, ±SEM.

To study the feeding activities of the WBPH, the electrical penetration graph (EPG) technique was used. There are five types of EPG signals based on the features of typical waves, which represent different insect activities described in materials and methods. The nonpenetration (NP) and xylem phase (N5) times were significantly increased after silencing of *SFMLP*, whereas the phloem-sap-ingestion phases were reduced significantly (N4-b) (Figure 4). The duration of the pathway phase (PP) or intracellular activity in the phloem (N4-a) have no significant differences between the *dsGFP* and *dsSFMLP* treatments (Figure 4).

### 2.4. SFMLP Is Necessary for Salivary Sheath Formation

**Figure 5:**
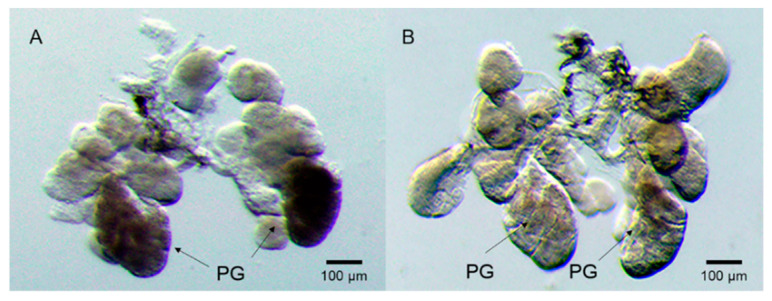
Effects of *SFMLP* knockdown on the morphology of the salivary gland of WBPH. (**A**) WBPH injected with *dsGFP*; (**B**) WBPH injected with *dsSFMLP*. PG indicates the principal gland. The arrow indicates the A-follicle of the principal gland.

Photographs of salivary gland morphology of the 5th nymphs were taken by a light stereomicroscope (Olympus SZX7). The morphologies of the WBPH salivary glands did not show significant differences between *dsGFP* and ds*SFMLP*. However, a colour change in the A-follicle of the principal gland was found between them. The A-follicle remained dark black in the *dsGFP*-treated WBPH (Figure 5A), but it appeared light in the *dsSFMLP*-treated WBPH after paraformaldehyde fixation (Figure 5B).

**Figure 6:**
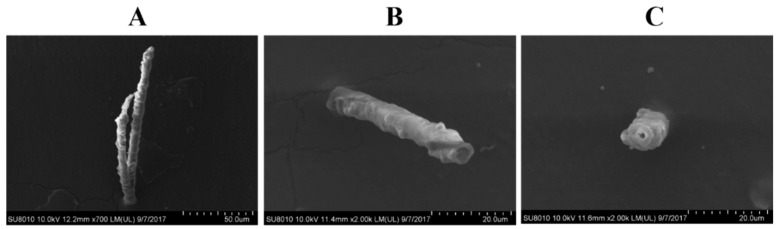
Effects of *SFMLP* knockdown on the morphology of the salivary sheath of WBPH (**A**) and (**B**) WBPH injected with *dsGFP*; (**C**) WBPH injected with *dsSFMLP*.

The salivary sheath formation was observed to further investigate the effects of *SFMLP* on feeding. After feeding on an artificial diet in Parafilm sachets for 1 day, WBPH salivary sheaths were analysed using scanning electron microscopy observation. The photographs revealed that WBPH subjected to *dsSFMLP* treatment produced significantly shorter and less-branched salivary sheaths than those produced by *dsGFP*-treated WBPH (Figure 6A–C).

**Figure 7:**
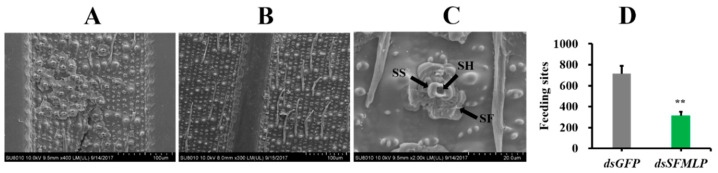
Effects of *SFMLP* knockdown on the probing trace of WBPH. (**A**) WBPH injected with *dsGFP*; (**B**) WBPH injected with *dsSFMLP*; (**C**) enlargement of the probing trace. SS, salivary sheath; SH, stylet hole; SF, salivary flange; (**D**) the flange number of WBPH injected with *dsSFMLP*. After feeding on rice for 24 h, the salivary sheaths were collected and observed under SEM. ** indicates statistically significant differences (*t-*test: *p* < 0.01). Bars, ±SEM.

We also used scanning electron microscopy to observe the penetration sites of WBPH on rice. On the same area of rice plants, the average number of feeding sites of *dsSFMLP*-treated WBPH was significantly lower than that of *dsGFP*-treated WBPH, and the numbers were 714 ± 76 and 314 ± 37, respectively (Figure 7A–D).

### 2.5. The Influences of SFMLP Silencing and Overexpression Mediated by Transgenic Rice on WBPH Performance

**Figure 8:**
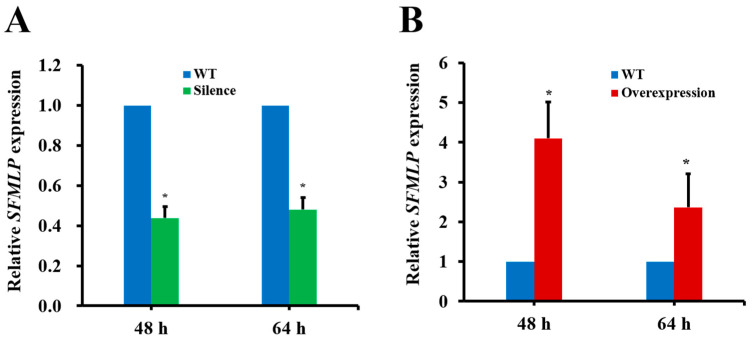
Relative expression levels of *SFMLP* in WBPHs feeding on transgenic plants. (**A**) *SFMLP* expression levels of WBPH feeding on *SFMLP*-*dsRNA*-transgenic plants (Silence) for 48 h and 64 h. (**B**) *SFMLP* expression levels of WBPH feeding on *SFMLP*-overexpressing transgenic plants (Overexpression) for 48 h and 64 h. * indicates statistically significant differences (*t-*test: *p* < 0.05). Bars, ±SEM.

To investigate the potential role of *SFMLP* as a plant-mediated transgenic target gene on WBPH management, transgenic rice plants with silenced and overexpressed *SFMLP* were generated. The transgenic (silencing and overexpression) and WT rice plants had no apparent phenotypic differences. The transcript levels of *SFMLP* were decreased by 52–57% in WBPH feeding on the *SFMLP*-silenced transgenic plants for 48 h and 64 h compared to those feeding on the WT plants (Figure 8A). In contrast, the transcript levels of *SFMLP* were increased by 136–310% in WBPH feeding on the *SFMLP*-overexpressing transgenic lines for 48 h and 64 h compared with those feeding on the WT plants (Figure 8B).

**Figure 9:**
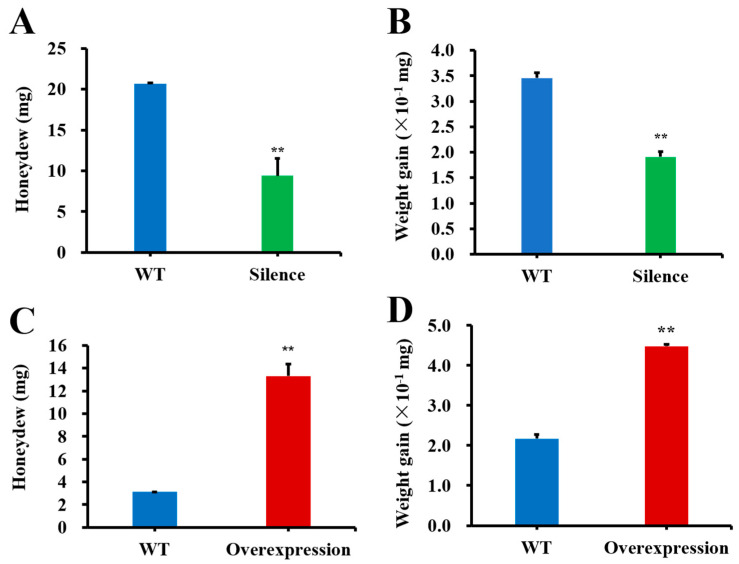
Effects on performance for WBPH feeding on transgenic plants. (**A**) Honeydew and (**B**) weight gains of WBPH feeding on *SFMLP*-*dsRNA*-transgenic plants (silence). (**C**) Honeydew and (**D**) weight gain of WBPH feeding on *SFMLP*-overexpressing transgenic plants (overexpression). ** indicates statistically significant differences (*t-*test: *p* < 0.01). Bars, ±SEM.

The honeydew excreted by WBPHs feeding on the *SFMLP*-silenced transgenic plants was decreased by 54.41% for the adults feeding for 72 h (Figure 9A). The body weights of the adults feeding on the *SFMLP*-silenced transgenic lines for 72 h were decreased by 44.76% (Figure 9B). In contrast, the honeydew excreted by WBPHs feeding on the *SFMLP*-overexpressing transgenic lines was increased by 324% for the adults feeding for 72 h (Figure 9C). The body weights of the adults feeding on the *SFMLP*-overexpressing transgenic lines for 72 h were increased by 106% (Figure 9D).

### 2.6. Transgenic Rice-Mediated Plant Defence Responses

**Figure 10:**
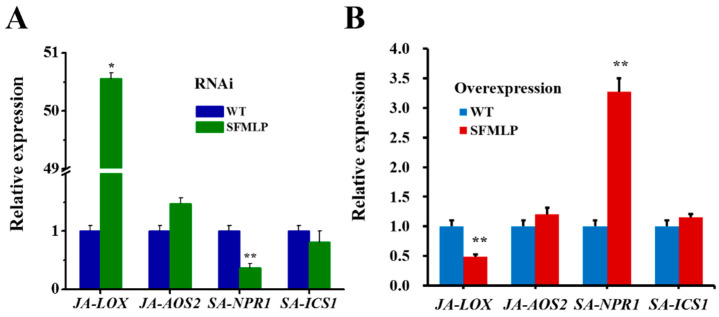
Relative expression levels of JA marker and SA marker genes in transgenic rice. (**A**) The expression levels of four genes in *SFMLP*-*dsRNA*-transgenic plants (*dsSFMLP*) and wild-type plants (WT); (**B**) the expression levels of four genes in *SFMLP*-overexpressing transgenic plants (*SFMLP*) and WT plants. * and ** indicate statistically significant differences (*t-*test: *p* < 0.05 and *p* < 0.01). Bars, ±SEM.

The relative expression levels of the SA marker genes (*NPR1* and *ICS1*) and the JA marker genes (*LOX* and *AOS2*) in the silenced and overexpressed transgenic rice plants showed significant differences from those of wild-type plants. In the *SFMLP-*silenced transgenic rice plants, they showed a lower expression level in SA pathway-*NPR1* and a higher expression level in JA pathway-*LOX* than those in wild-type plants (Figure 10A). In contrast, in *SFMLP*-overexpressing transgenic rice plants, the SA pathway-*NPR1* was expressed at a higher level, and the JA pathway-*LOX* showed a lower expression level than those in wild-type plants (Figure 10B).

**Figure 11:**
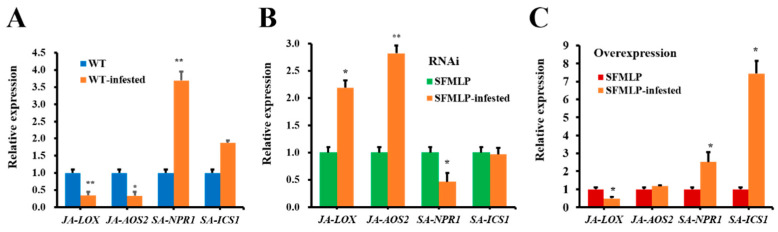
Relative expression levels of JA marker and SA marker genes in rice plants infested by WBPH. (**A**) The expression levels of genes in the WT plants and WT plants infested by WBPH (WT-infested); (**B**) the expression levels of genes in the *SFMLP*-*dsRNA*-transgenic plants (*dsSFMLP*) and *dsSFMLP* plants infested by WBPH (*SFMLP*-infested); (**C**) the expression levels of genes in the *SFMLP*-overexpressing-transgenic plants (*SFMLP*) and *SFMLP* plants infested by WBPH (*SFMLP*-infested). * and ** indicate statistically significant differences (*t-*test: *p* < 0.05 and *p* < 0.01). Bars, ±SEM.

After infestation by WBPH, in the wild-type plants, *NPR1* was expressed at higher levels than in those that were not infested, and *LOX* and *AOS2* were expressed at lower levels than in those that were not infested (Figure 11A). In the *SFMLP-*silenced transgenic rice plants, *LOX* and *AOS2* were expressed at higher levels than those in non-infested plants, and *NPR1* was expressed at lower levels than those in non-infested plants (Figure 11B). In the *SFMLP*-overexpressing transgenic rice plants, *NPR1* and *ICS1* were expressed at higher levels than those in non-infested plants, and *LOX* was expressed at lower levels than those in non-infested plants (Figure 11C).

## 3. Discussion

Mucin-like proteins have been studied to exist in many insects, such as in piercing-sucking planthoppers [8,33], leafhoppers [34], and several mosquito species [35]. The mucin-like proteins of the three major planthoppers, *S. furcifera, N. lugens,* and *L. striatellus*, are all rich in Ser with similar proportions (*S. furcifera* 22.8%, GenBank KX670544; *N. lugens* 22.4%, GenBank KY348750; *L. striatellus* 22.51%, GenBank JF502033), which are significantly different from those of *Nasonia vitripennis* (10.44%, GenBank NM_001130052) and *Holotrichia oblita* (4.57%, GenBank JF681187). It exhibits close ancestral relationships among the planthopper species compared with other insects. Ser can provide attachment sites for the formation of salivary sheaths. In aphids, the salivary protein SHP was found to aid the solidification of gelling saliva [36,37,38].

Our previous study revealed that *SFMLP* was highly expressed in the WBPH salivary gland, and the expression pattern of *SFMLP* was generally the same as that of mucin-like protein in *N. lugens* [8,33,39]. Mucin-like protein was found to be related to the salivary sheath formation of *N. cincticeps* and *N. lugens*, and feeding was affected significantly after mucin silencing [7,8,34,40]. In our study, the transcription of *SFMLP* was significantly reduced after its expression was knocked down, which indicates that introduction of double-stranded RNA (*dsRNA*) of *SFMLP* into WBPH causes the inhibition or degradation of the complementary mRNA of *SFMLP*. WBPHs silenced with *SFMLP* have incomplete, shorter, and less-branched salivary sheaths than those of control WBPH. As a result, the salivary sheaths of *SFMLP*-RNAi WBPH cannot reach the sieve tube, and WBPH will spend more time in the nonpenetration and pathway phases and less time feeding on the phloem. This decreased the amount of food intake, nymph mass, survival rate, and fecundity of the WBPH. In contrast, silence of *SFMLP* did not affect the ability of WBPH to feed on an artificial diet. These results suggest that SFMLP is necessary for WBPH to probe the rice plants and obtain the phloem sap. Based on the above results, it can be deduced that SFMLP is an essential component of the WBPH salivary sheath.

When feeding, herbivores secrete salivary effectors into host plant cells to elicit or suppress plant defence responses [1,2]. For instance, infestation by *N. lugens* can increase the levels of H_2_O_2_, SA, and ethylene but not JA in rice plants [41]. Mucin-like protein of *N. lugens* triggered rice defence responses associated with the JA signalling pathway revealed by the callose deposition maker [8] although endo-*β*-1,4-glucanase secreted by *N. lugens* did not elicit the rice defence-related signal molecules, including SA, JA, and jasmonoyl-isoleucine (JA-Ile) [5]. Wang et al. [42] found that the infestation of WBPH gravid females significantly increased the levels of JA, JA-Ile, and H_2_O_2_ and reduced the level of ethylene in rice although WBPH nymph infestation had no effect on these signal molecules. After infection by WBPH infected with the southern rice black-streaked dwarf virus, rice plant defences can partially be elicited, which is related to the smaller increase in SA and greater decrease in JA than nonviruliferous WBPH-infested plants [20]. In our study, after infestation by WBPH in rice, the relative expression levels of the SA marker gene *NPR1* were higher, and the JA marker genes *LOX* and *AOS2* were lower than those in wild-type plants. Overexpression of the *Bt56* gene in tobacco elicits the SA-signalling pathway and promotes the performance of the whitefly, whereas silencing the whitefly *Bt56* gene significantly decreases its feeding on host plants and makes it to lose the ability to induce the SA pathway [21]. Due to the salivary effector Bt56 being an SA elicitor, it promoted the whitefly performance on tobacco.

In this study, we obtained *SFMLP*-overexpressing and *SFMLP*-silenced transgenic plants to investigate their control effects on WBPH. For the overexpressed and silenced transgenic rice plants, the relative expression levels of the SA-signalling pathway and JA-signalling pathway genes showed the opposite results. WBPH infestation or not, overexpression of the *SFMLP* gene in planta elicits the SA signalling pathway corresponding to the upregulation of *NPR1* or *ICS1*, and silencing of the *SFMLP* gene suppresses the SA signalling pathway corresponding to the downregulation of *NPR1*. Therefore, from the above results, we propose that *SFMLP* triggers rice defence responses by eliciting SA and suppressing the JA signalling pathway. Furthermore, transgenic plant-mediated *SFMLP* RNAi decreased WBPH performance on rice plants and enhanced rice resistance to WBPH. In contrast, overexpression of *SFMLP* in rice promoted the feeding of WBPH on rice plants and impaired rice defences to WBPH. From the above results, we propose that overexpression of *SFMLP* in rice promoted WBPH performance because it activated the SA-signalling pathway. On the contrary, silence of *SFMLP* in rice decreased WBPH performance due to the repression of the SA-signalling pathway.

Host-induced gene silencing (HIGS) has great potential for effective control pest by generating transgenic plants carrying double-stranded *RNA*s (*dsRNA*s)-targeted genes against essential pest genes, and this technology is considered to the promising alternative to chemical pesticides [43]. The silence of *LsECP1* in *L. striatellus* decreases its fecundity and survival rates, and rice-mediated *LsECP1* knockdown enhanced rice resistance to *L. striatellus* [10]. When *Vg* of *L. striatellus* is overexpressed in rice plants, it can significantly hinder hydrogen peroxide (H_2_O_2_) accumulation and promote insect performance [9]. The fecundity of aphids was reduced after feeding on transgenic plants that were mediated by RNAi of salivary effector genes (*MpC002*, *MpPIntO1*, *Mp55*, *MpPIntO2*) [14,17,28,29,44]. Therefore, these effectors might be the potential plant-mediated RNAi targets for aphid control.

In summary, our results reveal that SFMLP plays dual roles as a component in WBPH sheath formation and activating the SA signalling pathway of rice defence responses. Furthermore, silencing or overexpressing *SFMLP* genes via transgenic rice plants can significantly influence WBPH performance in rice plants. This work not only deepens our knowledge of the physiological role of SFMLP and its effects on understanding plant-herbivore interactions but also provides a feasible strategy to control WBPH infestation by applying *SFMLP*-silenced transgenic rice under field conditions.

## 4. Materials and Methods

### 4.1. Plant Materials and Insects

Taichung Native 1 (TN1, a WBPH-susceptible rice cultivar), Kitaake (KIT, a WBPH-susceptible rice cultivar), and transgenic KIT lines with *SFMLP* overexpression or silencing were used in this work. All rice plants were grown within 100-mesh insect-proof cages (80 × 80 × 80 cm) in a greenhouse (30 ± 5 °C, 15 L: 9 D). WBPH colonies were originally obtained from rice fields in Xing’an County (Latitude 25.61° N, Longitude 110.67° E), Guilin City, Guangxi Zhuang Autonomous Region, China. The population was maintained on TN1 rice seedlings in a climate-controlled room at 27 ± 1 °C with a relative humidity (RH) of 75 ± 10% and a photoperiod of 16:8 (L:D).

### 4.2. RNAi Experiment

SFMLP in watery saliva of WBPH was identified by shotgun LC–MS/MS analysis, and the sequence of *SFMLP* from our transcriptomic analysis was similar to the full length of the WBPH mucin-like gene deposited in GenBank (accession number KX670544). In our previous study, *SFMLP* transcripts of WBPH were detected at higher levels in the salivary gland than in the head, gut, testes, ovary, and remaining body [39]. A 524 bp *SFMLP* fragment and a 594 bp control gene *GFP* fragment were amplified with primers harbouring a T7 promoter sequence (Table 1). *dsSFMLP* and *dsGFP* were synthesized using the HiScribe™ T7 Quick High Yield RNA Synthesis Kit (New England Biolabs, Ipswich, MA, USA) according to the manufacturer’s protocol. The *dsRNA* was resuspended in nuclease-free water to a concentration of 4 μg/μL and stored at −80 °C until use.

The 2nd or 5th instar WBPH nymphs (for details, see descriptions of different experiments) were anaesthetized with carbon dioxide for approximately 20 s and placed on 1% (*w*/*v*) agarose plates with their abdomens facing up for injection. A 25 nL (for the 2nd instar nymph) and 50 nL (for the 5th instar nymph) volume of *dsSFMLP* or *dsGFP* was injected into each insect at the junction between the prothorax and mesothorax using a microprocessor-controlled Nanolitre 2010 injector (World Precision Instruments). After injection, the injected WBPH were reared on rice cv TN1 plants for the following experiments.

### 4.3. Bioassay of WBPH Injected with dsRNA

To measure the survival rates of WBPH, the 2nd instar nymphs injected with *SFMLP* or *GFP dsRNA* were allowed to feed on TN1 plants or artificial diet. In the survival rate tests on rice plants, 20 WBPH nymphs were transferred to a glass tube (5 cm diameter × 40 cm height) with 1-month-old TN1 rice plants grown as described above, and the number of surviving nymphs was recorded daily for 10 days. For the survival rate tests on the artificial diet, a transparent plastic bottle (3 cm diameter × 5 cm height) was used as the feeding chamber, and the top lid was covered with sachets formed from two layers of stretched Parafilm (4 times the original area) containing 200 μL artificial diet with 2.5% sucrose solution. A 2% (*w*/*v*) agarose plate was placed at the bottom of the bottle to maintain humidity. Holes (6 mm in diameter) were made on the bottle body for ventilation. The artificial diet was changed once every 24 h. Twenty nymphs injected with *SFMLP* or *GFP dsRNA* were released into each bottle, and the number of surviving nymphs was recorded daily for 10 days. The survival rate tests on rice plants and on the artificial diet were both repeated five times.

To assess the effect of *SFMLP* knockdown on WBPH feeding, the 5th instar nymphs at 1 day after the injection of *SFMLP* or *GFP dsRNA* were starved for 1 h, and one nymph was transferred into a pre-weighed small Parafilm bag (4 × 4 cm), which was affixed to the rice stems. The nymph was transferred out after feeding for 24 h, and the Parafilm sachet was reweighed. The weight difference of the Parafilm bag was defined as the WBPH honeydew secretion. The experiment was replicated 25 times.

To investigate the influence of *SFMLP* knockdown on WBPH weight gain, 5th instar nymphs at 1 day after the injection of *SFMLP* or *GFP dsRNA* were weighed and allowed to feed on rice plants until new brachypterous adults emerged. The weight differences of the female and male adults (within 1 day of emergence) with the 5th instar nymphs were defined as female and male adult weight gain. The experiment was replicated 25 times.

### 4.4. EPG Recording of WBPH Feeding Behavior

The feeding behaviour of the WBPH was recorded using a Giga-8 direct current electrical penetration graph (DC-EPG) amplifier with a 10^9^-Ω input resistance in a Faraday cage (manufactured by Wageningen University, Wageningen, The Netherlands). The 5th instar WBPH nymphs at 1 day after the injection of *SFMLP* or *GFP dsRNA* were starved for 1 h before the EPG recordings. The WBPH attachment and amplifier connection methods were the same as those described by Lei et al. [45]. Each insect was continuously recorded for 6 h, and 10 to 15 replications were recorded in each treatment. Five distinctive output waveforms were interpreted with reference to the studies by Khan and Saxena [46] and Seo et al. [47], including NP for nonpenetration, PP (N1 + N2 + N3) for the pathway phase (including penetration initiation, salivation and stylet movement, and extracellular activity near the phloem), N4-a for intracellular activity in the phloem, N4-b for phloem sap ingestion, and N5 for the xylem phase. The EPG parameters were calculated for each recording with reference to Sarria et al. [48] and Lei et al. [45].

### 4.5. Observation of Salivary Glands and Salivary Sheaths of WBPH Injected with dsRNA

To assess the effects of *dsRNA* injection on the morphology of salivary glands, 5th instar WBPH nymphs at 1 day after the injection of *SFMLP* or *GFP dsRNA* were allowed to feed on TN1 rice until the emergence of adults. The salivary glands of adults within 1 day of emergence were dissected using micro forceps (Shanghai Medical Instruments Ltd., Corp., Shanghai, China) in 1 × phosphate buffered solution and transferred into 4% paraformaldehyde buffer for fixation for 30 min. The morphology of the salivary gland was observed under an anatomical lens (Leica Microsystems GmbH, Wentzler, Germany). The experiment was replicated 10 times.

To explore the effects of *dsRNA* injection on the morphology of salivary sheaths, the 5th instar WBPH nymphs injected with *SFMLP* or *GFP dsRNA* were allowed to feed on TN1 rice for 1 day, and 30 nymphs were transferred into the feeding chamber, which was a transparent plastic bottle (5 cm long and 3 cm in diameter) with the top lid covered with sachets formed from two layers of stretched Parafilm (4 times the original area) containing 200 μL 2.5% sucrose solution. A 2% (*w*/*v*) agarose plate was placed at the bottom to maintain humidity, and holes (6 mm in diameter) were made on the bottle body for ventilation. After feeding for 1 day, the inner Parafilm layer was washed three times using 1 × phosphate-buffered solution and dried in a vacuum desiccator for morphological observation using scanning electron microscopy. The experiment was replicated three times.

To observe the feeding frequencies, the 5th instar WBPH nymphs injected with *SFMLP* or *GFP dsRNA* were allowed to feed on TN1 rice for 1 day, and 30 nymphs were transferred into the feeding chamber, which was a plastic tube (4 cm diameter and 6 cm long) affixed to the base of a rice stem and covered by sponges at both ends, leaving an approximately 4 cm long feeding space inside. After feeding for 1 day, the feeding site was collected and fixed with 4% glutaraldehyde for 48 h and then 1% osmium tetroxide for 3 h. After washing and dehydration, the feeding sites were observed using scanning electron microscopy. The experiment was replicated three times.

### 4.6. Transgenic Rice Plant Development and the WBPH Bioassay

To obtain the *SFMLP*-silenced transgenic rice lines, the 250 bp cDNA fragment was inserted into a Gateway pENTR/D-TOPO cloning vector, which carried two recombination sites for the LR Clonase reaction. Then, the fragment derived from a target gene was transferred into a pANDA destination vector to generate a hairpin RNAi. To obtain the *SFMLP*-overexpressing transgenic rice lines, the full CDS region containing a 2160 bp cDNA fragment was inserted into the PRHV cloning vector. The final RNAi and overexpression vectors were introduced into *Agrobacterium tumefaciens* (strain EHA105) employing the heat-shock method. The construct was transformed into the rice plants of the variety KIT via an *Agrobacterium tumefaciens*-mediated method. Transgenic rice plants, either overexpressing or silencing *SFMLP*, were developed by the Institute of Crop Sciences, Chinese Academy of Agricultural Sciences. Integration of the *SFMLP* fragment in transgenic rice lines was determined via PCR and quantitative real-time PCR (RT–qPCR) (Figures S1 and S2). The primers used for *dsMucin* detection are listed in Table 1. The target plants were screened according to the above methods for subsequent experiments.

To investigate the *SFMLP* expression level of WBPH feeding on silenced and overexpression transgenic rice plants, single 1-month-old rice plants (transgenic rice lines and wild-type plants) were planted in one plastic pot (8 cm diameter and 27 cm height) and maintained as described above. A total of ten 5th instar WBPH nymphs were released into pots and transferred out after feeding for 48 h or 64 h for RNA isolation and cDNA synthesis. The cDNA samples were subjected to RT–qPCR to detect the expression level of the *SFMLP* gene. The experiment was repeated three times.

To assess the effect of *SFMLP* silencing or overexpression in rice plants on WBPH performance, 1-month-old rice plants (transgenic rice lines and wild-type plants) were planted in plastic pots (8 cm diameter and 13.5 cm height). After starvation for 1 h, the adults (within 1 day of emergence) were weighed, and then, one adult was transferred into a pre-weighed small Parafilm bag (4 × 4 cm), which was affixed to the rice stems. The adult was transferred out after feeding for 72 h, and the adult and Parafilm sachets were reweighed. The differences in the two weights for Parafilm bags and adults were defined as WBPH honeydew secretion and weight gain, respectively. The experiment was replicated 25 times.

### 4.7. Analysis of the Relative Expression of Phytohormone-Related Genes

To quantify the transcript levels of phytohormone-related genes in leaf sheaths of transgenic and wild-type rice plants infested by WBPH, 1-month-old rice plants were planted in plastic pots (8 cm diameter and 27 cm height). After starvation for 1 h, a total of twenty-five 5th instar nymphs were released into each pot and maintained as described above. Rice stems (5–10 cm) from the middle parts of the leaf sheath were collected from each pot, and then, all samples were ground in liquid nitrogen for RNA isolation and cDNA synthesis. The experiment was repeated four times.

The transcript levels of SA- and JA-related genes in rice leaf sheaths were quantified using RT–qPCR for the three treatment groups (Table 1). The extraction of total RNA from the leaf sheath samples and the synthesis of cDNA were described by Li et al., 2016 [40]. qPCR was performed using the Bester SybrGreen qPCR mastermix (DBI Bioscience, Germany) with the 7500 Sequence Detection System (Applied Biosystems, Foster City, CA, USA). Amplification reactions were performed in a 20 μL final volume containing 10 μL of TB Green qPCR premix Ex Taq II, 0.8 μL of forward primer (10 μM) and reverse primer (10 μM) pairs (Table 1), 0.4 μL of Rox II, 5 μL of cDNA (100 ng/μL), and 3 μL of sterilized H_2_O. The reaction conditions were as follows: 95 °C for 30 s followed by 40 cycles of 5 s at 95 °C and 34 s at 60 °C, followed by melt curve stages. Blank controls without template were included in each experiment. qPCR was performed for three samples, and each sample had three technical repeats. The comparative 2^−^^ΔΔ^^CT^ method was used to calculate the relative gene expression levels in different samples [49].

### 4.8. Statistical Analysis

All the results were based on the mean values excluding the morphology figure. The statistical significance of differences between treatments and control groups was assessed by Student’s *t-*test (* *p* < 0.05, ** *p* < 0.01). The error bars represent the means ± standard errors (SE). All analyses were conducted in IBM SPSS Statistics version 22.0 (SPSS Inc., Chicago, IL, USA).

## Figures and Tables

**Table 1:** The primers used in this study.

Primers	Primer Sequence (5′-3′)	
**qPCR**		
*mucin*-*F*	TCATCTTCTGGTGTGCATTC	
*mucin*-*R*	GGGGGGTGTTGAAAATAGTA	
*L9-F*	CAAGATGAGAGCCGTGTA	
*L9-R*	CGAGTTGGTAACAGTGAC	
*L10-F*	GCGACTTCATCCGTTCCA	
*L10-R*	CACTCTAGCCACTGTTCCTT	
*SA-NPR1-F*	TTTCCGATGGAGGCAAGAG	
*SA-NPR1-R*	GCTGTCATCCGAGCTAAGTGTT	
*JA-LOX-F*	GCATCCCCAACAGCACATC	
*JA-LOX-R*	AATAAAGATTTGGGAGTGACATA	
**RNAi**		
*dsmucin-T7-F*	TAATACGACTCACTATAGGGGTACTACCCAAAACTCTCCCAAATC	
*dsmucin-T7-R*	TAATACGACTCACTATAGGGGCAAGTGCCTGAAGAACAATGAAG	
*dsGFP-T7-F*	TAATACGACTCACTATAGGGGGAGAAGAACTTTTCACTGG	
*dsGFP-T7*-*R*	TAATACGACTCACTATAGGGAGTTGAACGGATCCATCTTC	
**Expression in plant**		
*Overexpression-Mucin-F*	CCG gatatc ATGAGGTGTTTCTCAGTTATCG	
*Overexpression-Mucin-R*	ATA actagt CCAGGCACTGTAACCACCTC	
*dsmucin-F*	GGATCCtctctgctgcatcatgctacgg	
*dsmucin-R*	CTCGAGccataagctccaccagctgc	

## Data Availability

The data that support the findings of this study are available from the corresponding author upon request.

## Supplementary Materials

The following supporting information can be downloaded at: https://www.mdpi.com/article/10.3390/ijms23158239/s1.

## References

[1] Felton G.W., Chung S.H., Hernandez M.G.E., Louis J., Peiffer M., Tian D. (2014). Herbivore oral secretions are the first line of protection against plant-induced defences. Annu. Plant Rev..

[2] Miles P.W. (1999). Aphid saliva. Biol. Rev. Camb. Philos. Soc..

[3] Hogenhout S.A., Van der Hoorn R.A., Terauchi R., Kamoun S. (2009). Emerging concepts in effector biology of plant-associated organisms. Mol. Plant Microbe Interact..

[4] Hao P., Liu C., Wang Y., Chen R., Tang M., Du B., Zhu L., He G. (2008). Herbivore-induced callose deposition on the sieve plates of rice: An important mechanism for host resistance. Plant Physiol..

[5] Ji R., Ye W., Chen H., Zeng J., Li H., Yu H., Li J., Lou Y. (2017). A salivary endo-*β*-1,4-glucanase acts as an effector that enables the brown planthopper to feed on rice. Plant Physiol..

[6] Ye W., Yu H., Jian Y., Zeng J., Ji R., Chen H., Lou Y. (2017). A salivary EF-hand calcium-binding protein of the brown planthopper *Nilaparvata lugens* functions as an effector for defense responses in rice. Sci. Rep..

[7] Huang H.J., Liu C.W., Huang X.H., Zhou X., Zhuo J.C., Zhang C.X., Bao Y.Y. (2016). Screening and functional analyses of *Nilaparvata lugens* salivary proteome. J. Proteome Res..

[8] Shangguan X., Zhang J., Liu B., Zhao Y., Wang H., Wang Z., Guo J., Rao W., Jing S., Guan W. (2018). A mucin-like protein of planthopper is required for feeding and induces immunity response in plants. Plant Physiol..

[9] Ji R., Fu J., Shi Y., Li J., Jing M., Wang L., Yang S., Tian T., Wang L., Ju J. (2021). Vitellogenin from planthopper oral secretion acts as a novel effector to impair plant defenses. New Phytol..

[10] Tian T., Ji R., Fu J., Li J., Wang L., Zhang H., Yang S., Ye W., Fang J., Zhu-Salzman K. (2021). A salivary calcium-binding protein from *Laodelphax striatellus* acts as an effector that suppresses defense in rice. Pest Manag. Sci..

[11] Huang H.J., Cui J.R., Xia X., Chen J., Ye Y.X., Zhang C.X., Hong X.Y. (2019). Salivary DNase II from *Laodelphax striatellus* acts as an effector that suppresses plant defence. New Phytol..

[12] Atamian H.S., Chaudhary R., Cin V.D., Bao E., Girke T., Kaloshian I. (2013). In planta expression or delivery of potato aphid *Macrosiphum euphorbiae* effectors *Me10* and *Me23* enhances aphid fecundity. Mol. Plant Microbe Interact..

[13] Bos J.I., Prince D., Pitino M., Maffei M.E., Win J., Hogenhout S.A. (2010). A functional genomics approach identifies candidate effectors from the aphid species *Myzus persicae* (green peach aphid). PLoS Genet..

[14] Elzinga D.A., De Vos M., Jander G. (2014). Suppression of plant defenses by a *Myzus persicae* (green peach aphid) salivary effector protein. Mol. Plant Microbe Interact..

[15] Guo H., Zhang Y., Tong J., Ge P., Wang Q., Zhao Z., Zhu-Salzman K., Hogenhout S.A., Ge F., Sun Y. (2020). An aphid-secreted salivary protease activates plant defense in phloem. Curr. Biol..

[16] Mutti N.S., Louis J., Pappan L.K., Pappan K., Begum K., Chen M.S., Park Y., Dittmer N., Marshall J., Reese J.C. (2008). A protein from the salivary glands of the pea aphid, *Acyrthosiphon pisum*, is essential in feeding on a host plant. Proc. Natl. Acad. Sci. USA.

[17] Pitino M., Hogenhout S.A. (2013). Aphid protein effectors promote aphid colonization in a plant species-specific manner. Mol. Plant. Microbe Interact..

[18] Walling L.L. (2008). Avoiding effective defenses: Strategies employed by phloem-feeding insects. Plant Physiol..

[19] Zhou G., Qi J., Ren N., Cheng J., Erb M., Mao B., Lou Y. (2009). Silencing *OsHI-LOX* makes rice more susceptible to chewing herbivores, but enhances resistance to a phloem feeder. Plant J..

[20] Li P., Liu H., Li F., Liao X., Ali S., Hou M. (2018). A virus plays a role in partially suppressing plant defenses induced by the viruliferous vectors. Sci. Rep..

[21] Xu H.X., Qian L.X., Wang X.W., Shao R.X., Hong Y., Liu S.S., Wang X.W. (2019). A salivary effector enables whitefly to feed on host plants by eliciting salicylic acid-signaling pathway. Proc. Natl. Acad. Sci. USA.

[22] Moran P.J., Thompson G.A. (2001). Molecular responses to aphid feeding in Arabidopsis in relation to plant defense pathways. Plant Physiol..

[23] Zarate S.I., Kempema L.A., Walling L.L. (2007). Silverleaf whitefly induces salicylic acid defenses and suppresses effectual jasmonic acid defenses. Plant Physiol..

[24] Zhang P.J., Li W.D., Huang F., Zhang J.M., Xu F.C., Lu Y.B. (2013). Feeding by whiteflies suppresses downstream jasmonic acid signaling by eliciting salicylic acid signaling. J. Chem. Ecol..

[25] Alon M., Malka O., Eakteiman G., Elbaz M., Moyal Ben Zvi M., Vainstein A., Morin S. (2013). Activation of the phenylpropanoid pathway in *Nicotiana tabacum* improves the performance of the whitefly *Bemisia tabaci* via reduced jasmonate signaling. PLoS ONE.

[26] Francischetti I.M., Valenzuela J.G., Pham V.M., Garfield M.K., Ribeiro J.M. (2002). Toward a catalog for the transcripts and proteins (sialome) from the salivary gland of the malaria vector *Anopheles gambiae*. J. Exp. Biol..

[27] Thakur N., Upadhyay S.K., Verma P.C., Chandrashekar K., Tuli R., Singh P.K. (2014). Enhanced whitefly resistance in transgenic tobacco plants expressing double stranded RNA of *v-ATPase A* gene. PLoS ONE.

[28] Abdellatef E., Will T., Koch A., Imani J., Vilcinskas A., Kogel K.H. (2015). Silencing the expression of the salivary sheath protein causes transgenerational feeding suppression in the aphid *Sitobion avenae*. Plant Biotechnol. J..

[29] Pitino M., Coleman A.D., Maffei M.E., Ridout C.J., Hogenhout S.A. (2011). Silencing of aphid genes by dsRNA feeding from plants. PLoS ONE.

[30] Sun Y., Sparks C., Jones H., Riley M., Francis F., Du W., Xia L. (2019). Silencing an essential gene involved in infestation and digestion in grain aphid through plant-mediated RNA interference generates aphid-resistant wheat plants. Plant Biotechnol. J..

[31] Yang J., Sun X.Q., Zhu-Salzman K., Qin Q.M., Feng H.Q., Kong X.D., Zhou X.G., Cai Q.N. (2020). Host-induced gene silencing of brown planthopper glutathione S-transferase gene enhances rice resistance to sap-sucking insect pests. J. Pest Sci..

[32] Zha W., Peng X., Chen R., Du B., Zhu L., He G. (2011). Knockdown of midgut genes by dsRNA-transgenic plant-mediated RNA interference in the hemipteran insect *Nilaparvata lugens*. PLoS ONE.

[33] Huang H.J., Liu C.W., Xu H.J., Bao Y.Y., Zhang C.X. (2017). Mucin-like protein, a saliva component involved in brown planthopper virulence and host adaptation. J. Insect Physiol..

[34] Hattori M., Komatsu S., Noda H., Matsumoto Y. (2015). Proteome analysis of watery saliva secreted by green rice leafhopper, *Nephotettix cincticeps*. PLoS ONE.

[35] Das S., Radtke A., Choi Y.J., Mendes A.M., Valenzuela J.G., Dimopoulos G. (2010). Transcriptomic and functional analysis of the *Anopheles gambiae* salivary gland in relation to blood feeding. BMC Genom..

[36] Korayem A.M., Fabbri M., Takahashi K., Scherfer C., Lindgren M., Schmidt O., Ueda R., Dushay M.S., Theopold U. (2004). A *Drosophila* salivary gland mucin is also expressed in immune tissues: Evidence for a function in coagulation and the entrapment of bacteria. Insect Biochem. Mol. Biol..

[37] Carolan J.C., Fitzroy C.I., Ashton P.D., Douglas A.E., Wilkinson T.L. (2009). The secreted salivary proteome of the pea aphid *Acyrthosiphon pisum* characterised by mass spectrometry. Proteomics.

[38] Will T., Vilcinskas A. (2015). The structural sheath protein of aphids is required for phloem feeding. Insect Biochem. Mol. Biol..

[39] Miao Y.T., Deng Y., Jia H.K., Liu Y.D., Hou M.L. (2018). Proteomic analysis of watery saliva secreted by white-backed planthopper, *Sogatella furcifera*. PLoS ONE.

[40] Liu X., Zhou H., Zhao J., Hua H., He Y. (2016). Identification of the secreted watery saliva proteins of the rice brown planthopper, *Nilaparvata lugens* (Stål) by transcriptome and Shotgun LC-MS/MS approach. J. Insect Physiol..

[41] Wang X., Zhou G., Xiang C., Du M., Cheng J., Liu S., Lou Y. (2008). *β*-Glucosidase treatment and infestation by the rice brown planthopper *Nilaparvata lugens* elicit similar signaling pathways in rice plants. Chin. Sci. Bull..

[42] Wang W., Yu Z., Meng J., Zhou P., Luo T., Zhang J., Wu J., Lou Y. (2020). Rice phenolamindes reduce the survival of female adults of the white-backed planthopper *Sogatella furcifera*. Sci. Rep..

[43] Kong L.A., Shi X., Chen D., Yang N., Yin C.F., Yang J., Wang G.F., Huang W.K., Peng H., Peng D.L. (2022). Host-induced silencing of a nematode chitin synthase gene enhances resistance of soybeans to both pathogenic *Heterodera glycines* and *Fusarium oxysporum*. Plant Biotechnol. J..

[44] Coleman A.D., Wouters R.H., Mugford S.T., Hogenhout S.A. (2015). Persistence and transgenerational effect of plant-mediated RNAi in aphids. J. Exp. Bot..

[45] Lei W., Li P., Han Y., Gong S., Yang L., Hou M. (2016). EPG recordings reveal differential feeding behaviors in *Sogatella furcifera* in response to plant virus infection and transmission success. Sci. Rep..

[46] Khan Z.R., Saxena R.C. (1984). Electronically recorded waveforms associated with the feeding behavior of *Sogatella furcifera* (Homoptera: Delphacidae) on susceptible and resistant rice varieties. J. Econ. Entomol..

[47] Seo B.Y., Kwon Y.H., Jung J.K., Kim G.H. (2009). Electrical penetration graphic waveforms in relation to the actual positions of the stylet tips of *Nilaparvata lugens* in rice tissue. J. Asia-Pac. Entomol..

[48] Sarria E., Cid M., Garzo E., Fereres A. (2009). Excel workbook for automatic parameter calculation of EPG data. Comput. Electron. Agric..

[49] Livak K.J., Schmittgen T.D. (2001). Analysis of relative gene expression data using real-time quantitative PCR and the 2^-∆∆CT^ method. Methods.

