# Supplementary material for: Sogatella furcifera Saliva Mucin-like Protein Is Required for Feeding and Induces Rice Defences
Source: Int J Mol Sci. 2022 Jul 26;23(15):8239. doi: 10.3390/ijms23158239 (PMC9332473; doi:10.3390/ijms23158239)
Supplement: Supplementary file 1 [file ijms-23-08239-s001.zip › ijms-1818264-supplementary.pdf]

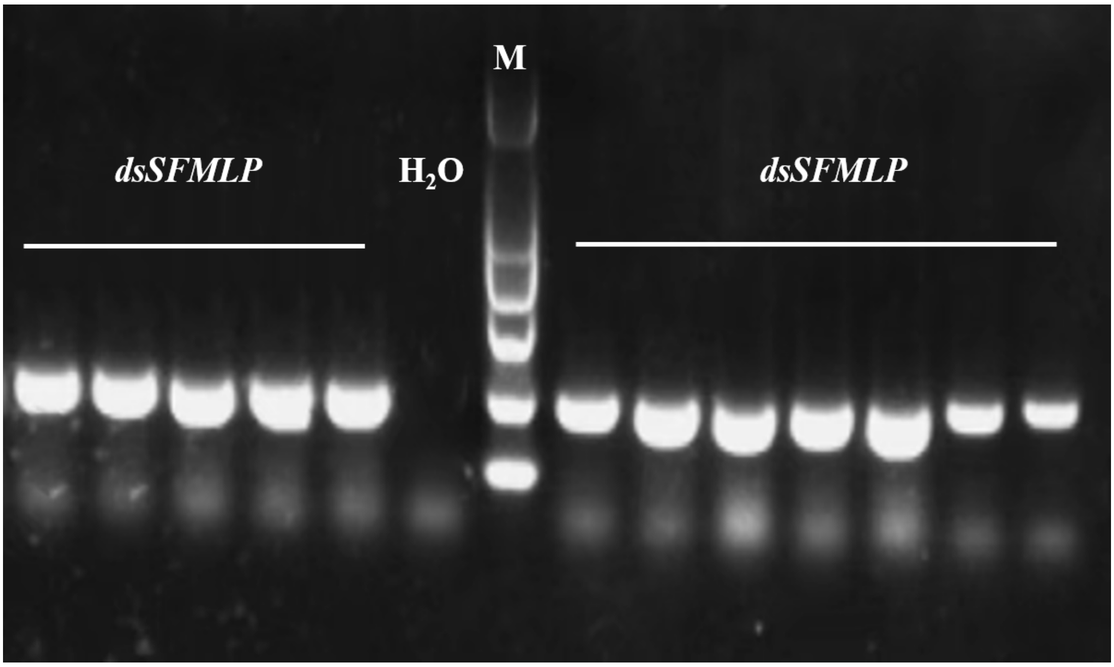

Figure S1. Detection of *dsSFMLP* fragment in *SFMLP*-silenced transgenic rice lines via PCR. M, DNA marker in this lane; H_2O, blank control (no template DNA) in this lane; *dsSFMLP*, *dsSFMLP* fragment amplified from the genome DNAs of *SFMLP*-silenced transgenic rice lines in these lanes.

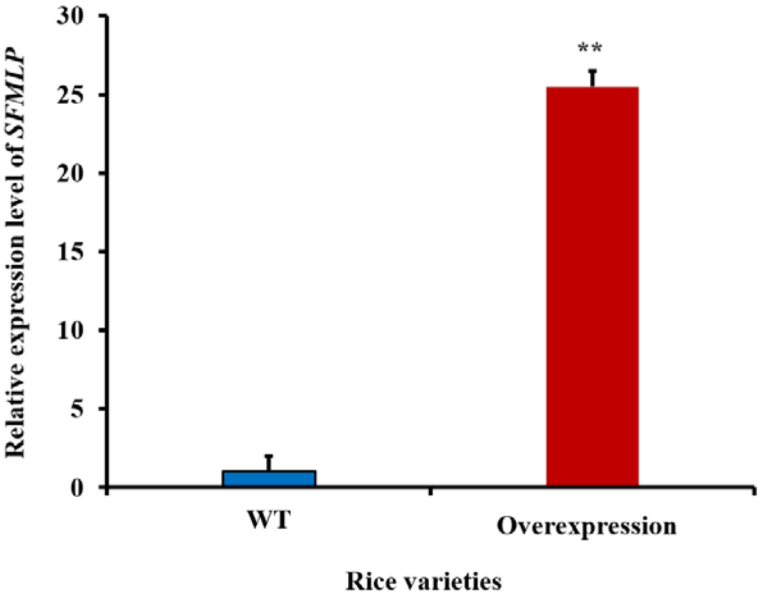

Figure S2. Detection of *SFMLP*-overexpressing transgenic rice lines via quantitative real-time PCR (RT–qPCR). WT, wild-type plants; Overexpression, *SFMLP*-overexpressing transgenic rice lines. ** indicates statistically significant differences (*t*-test: $p < 0.01$). Bars, ± SEM.
